# Bilateral optic nerve infiltration and leukemic retinopathy as initial signs of leukemia relapse with central nervous system involvement in an adult: a case report

**DOI:** 10.1186/s12886-024-03486-7

**Published:** 2024-05-28

**Authors:** Yisai Wang, Ling Zhu, Hongtao Wang, Zhen Li, Ruomeng Bai, Qinghua Wei, Lin Huang, Ying Xu, Songguo Li, Hui Chen

**Affiliations:** 1 Department of Ophthalmology, Peking University Shougang Hospital, 9# Jinyuanzhuang Road, Shijingshan district, Beijing, 100144 China; 2 Department of haematology, Air Force Medical University, Beijing, China

**Keywords:** Case report, Philadelphia chromosome-positive acute lymphoblastic leukemia, Optic nerve infiltration, Leukemic retinopathy, Relapse

## Abstract

**Background:**

We describe a case in which bilateral optic nerve infiltration and leukemic retinopathy were the initial signs of disease relapse in a patient with Philadelphia chromosome-positive acute lymphoblastic leukemia (Ph^+^-ALL) with central nervous system (CNS) involvement.

**Case presentation:**

A 65-year-old Asian female with Ph^+^-ALL in complete remission presented at our institution with symptoms of visual disturbance, central scotoma and pain with eye movement in both eyes for a 1-month duration. Ophthalmic examination revealed remarkable optic disc swelling with multiple flame-shaped peripapillary hemorrhages, retinal venous dilation and retinal hemorrhages in both eyes. She was subsequently referred to the treating oncologist and diagnosed with Ph^+^-ALL relapse with multiple relapsed diseases involving the bone marrow and CNS. After intrathecal (IT) therapy, her visual acuity dramatically improved, and her leukemic infiltrates decreased.

**Conclusions:**

To the best of our knowledge, this is the first case report of ALL relapse with CNS involvement presenting as bilateral optic nerve infiltration and leukemic retinopathy in an adult. Hence, we highlight the priority and sensitivity of ophthalmic examinations, as they are noninvasive methods for detecting leukemia relapse.

## Background

Leukaemia is a malignant haematologic disease caused by acquired clonal abnormalities of haematopoietic stem cells that replace normal bone marrow, and acute lymphoblastic leukaemia (ALL) accounts for approximately 70% of all cases of this disease [1]. Most adults with acute ALL who achieve complete remission (CR) will relapse, whatever their prior treatment, cannot be rescued using currently available therapies [2]. The central nervous system (CNS) is the most important site of extramedullary disease in adults with acute lymphoblastic leukemia (ALL). There have been few studies among adult patients focused on CNS disease [3], and prevention of recurrence is the best strategy for long-term survival in patients with this disease [4]. The Philadelphia (Ph) chromosome is the most common cytogenetic abnormality found in adult B-cell lineage ALL [5] and has been proven to be an adverse prognostic factor in ALL [6]. The clinical presentation of Philadelphia chromosome-positive acute lymphoblastic leukemia (Ph^+^-ALL), similar to other forms of ALL, is largely nonspecific [7]. Although patients with leukemia classically present with fatigue, fever and bleeding, ocular manifestations occur in up to 90% of these patients [8]. All parts of the eye can be involved either prior to systemic diagnosis of leukemia or during the disease course [9], with manifestations relating to direct leukemic infiltration or from secondary effects from hematologic abnormalities, CNS involvement or opportunistic infections [10].

In the literature, few reports of visual symptoms as the first sign of ALL relapse have been published. We describe a rare case in which an elderly female presented with bilateral blurry vision as the first sign of Ph^+^-ALL relapse. This is the first report of bilateral optic nerve infiltration and leukemic retinopathy as initial signs of ALL relapse with CNS involvement in an adult.

## Case presentation

**Fig. 1:**
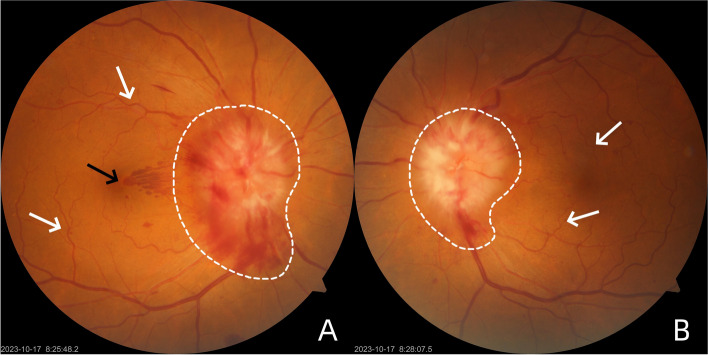
Fundus photos of both eyes at the presentation. **A** Fundus photo of the RE showed a dramatic creamy-white optic nerve infiltration with multiple flame-shaped peripapillary hemorrhages (white outlines), retinal venous dilation and tortuosity (white arrows), and blot hemorrhages on the foveal area (black arrows). **B** Fundus photo of the LE with optic disc edema and peripapillary hemorrhages (white outlines), retinal venous dilation and tortuosity (white arrows), less pronounced than RE

**Fig. 2:**
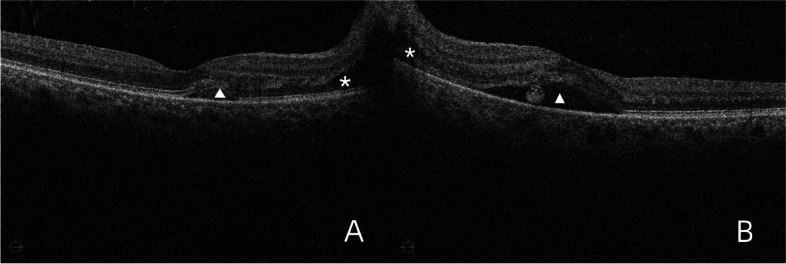
Optical coherence tomography (OCT) at the presentation. **A** OCT of the RE revealed exudates with irregularity in retinal layers (triangle) and subretinal fluid around the optic disc (asterisk). **B** OCT of the LE showed serous macular detachments with subretinal hyperreflectivity (triangle) and subretinal fluid around the optic disc (asterisk)

**Fig. 3:**
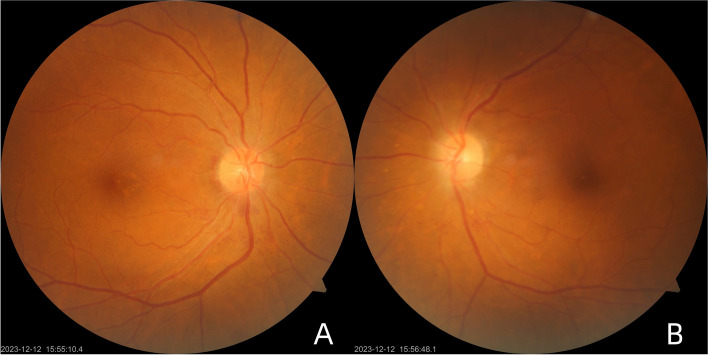
Fundus photos of both eyes taken 8 weeks after the initial presentation. **A** Fundus photo of the RE showed an absorption in macular and peripheral hemorrhage. **B** The disc margins were slightly blurry and vascular tortuosity was less pronounced in the LE

**Fig. 4:**
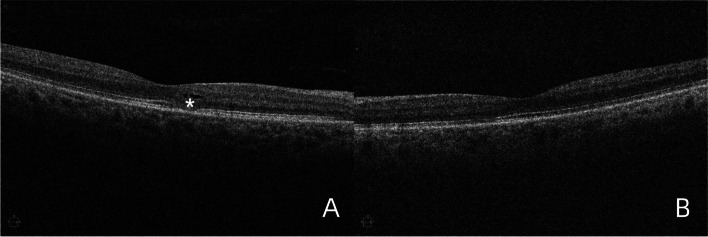
OCT taken 8 weeks after the initial presentation. **A **OCT of the RE revealed the persistent rupture of Ellipsoid Zone(asterisk). **B **OCT of the LE showed subretinal fluid disappeared basically

A 65-year-old Asian female with Ph^+^-ALL in complete remission presented at our institution with symptoms of visual disturbance, central scotoma and pain with eye movement in both eyes for a 1-month duration. Her past medical history included a diagnosis of Ph^+^-ALL initially presenting as fever 2 years previously, after which she started chemotherapy for 18 months. At the time of presentation, her ALL status was in complete remission, and her laboratory test results were normal for 6 months. Ocular examination revealed a best-corrected visual acuity (BCVA) of 20/40 in the right eye (RE) and 20/50 in the left eye (LE). The intraocular pressure (IOP) of both eyes was within normal limits. Both eyes had normal pupillary reflexes and normal ocular adnexa, a quiet anterior chamber, a clear lens with increased density and no vitreous cells in the anterior vitreous face. Overall, the anterior segment examination was unremarkable. A fundus photo showed dramatic creamy-white optic nerve infiltration with multiple flame-shaped peripapillary hemorrhages, retinal venous dilation and tortuosity, and blot hemorrhages on the foveal area in the RE (Fig. 1A). The LE had a similar hemorrhagic fundal presentation as the RE, except that fewer peripapillary and retinal hemorrhages were located in the LE (Fig. 1B). Optical coherence tomography (OCT) revealed exudates with irregularity in retinal layers and subretinal fluid around the optic disc in the RE (Fig. 2A). Serous macular detachments with subretinal hyperreflectivity and subretinal fluid around the optic disc were observed in LE (Fig. 2B). Neither neurologic nor dermatologic signs were present on physical examination. Based on the ocular findings and history of blood disease, the patient was referred to the treating oncologist for further management. Peripheral blood investigations (performed within 3 days of initial presentation) revealed a hemoglobin level of 11.6 g/dL, a white blood cell count of 3680/µL, and a platelet count of 129,000/µL. Bone marrow examination revealed an increase in blast cells. Lumbar puncture (LP) showed normal cerebrospinal fluid (CSF) open pressure (16 cm). Flow cytometry analysis of CSF samples revealed 1667/µL nucleated cells and 97% immature B cells with a phenotype similar to that of cells detected at initial diagnosis. Cerebral magnetic resonance imaging (MRI) showed no enhancement of the optic nerve top or bottom. She was managed as a case of ALL relapse with CNS involvement and the patient was subsequently treated with 12 cycles of intrathecal (IT) therapy (methotrexate, dexamethasone, and cytarabine) for 6 weeks. Cranial irradiation was scheduled but delayed for personal reasons. We followed up with the patient for a duration of 8 weeks, during which she experienced visual recovery, as revealed by a BCVA of 20/25 in the RE and 20/32 in the LE. However, complete resolution has not yet been achieved. Follow-up fundus examination (Fig. 3) along with OCT (Fig. 4) revealed that retinal hemorrhages were significantly reduced and that edema of the optic nerve decreased in both eyes.

## Discussion and conclusions

The presence of specific ocular lesions was associated with a higher frequency of bone marrow relapses and CNS involvement, leading to a lower survival rate [11]. Leukemic retinopathy is the term most often used to denote the fundus manifestations of anaemia, thrombocytopenia and hyperviscosity observed in patients with leukaemia [12]. Experts believe that leukemic retinopathy is associated with abnormal hematologic parameters and coagulopathy [13, 14]. Our patient presented with bilateral retinal vein dilatation and tortuosity, blot and flame-shaped retinal hemorrhages, and serous retinal detachment, features that were the result of both direct retinal leukemic infiltration and leukostasis secondary to hyperleukocytosis [15]. However, serous retinal detachment (SRD) in her LE has been reported less commonly in the setting of acute leukemia and may develop as a result of choroidal involvement by leukemic cells causing retinal pigment epithelial disturbances or due to incompetence of the outer blood‒retinal barrier (BRB), which induces retinal pigment epithelial changes [16]. Although they are more common in acute (vs. chronic) leukemia, they are less common in patients with the lymphoid (vs. myeloid) subtypes, females and older patients, making our patient an unlikely suspect [17]. With the increase in survival of patients with leukemia, the incidence of CNS relapse is on the rise. The optic nerve can reveal leukemic involvement of the CNS [18]. However, optic nerve infiltration as an initial sign of relapse is quite rare. The eyes and optic nerves are identified as pharmacological sanctuaries for leukemia therapy because the BRB shields the CNS from any systemically administered chemotherapeutic drugs [19]. Thus, many adjuvant therapies are often required to achieve complete remission. In our patient, bone marrow and CSF aspiration showed massive leukemic cells, while the CSF open pressure was normal. Optic neuropathy in the context of leukemia can be diagnosed through a broad variety of methods, including infiltration, infections, compression, vasculitis, radiotherapy-induced complications or adverse effects of chemotherapeutic agents [20–22]. All these possibilities should be excluded through careful investigations. There were no signs of infection. Furthermore, the absence of neurologic and dermatologic signs, such as dysacusis, vitiligo, and poliosis, is not consistent with Harada’s syndrome. A diagnosis of leukemic optic nerve infiltration was highly considered. An 8-week follow-up appointment at the ophthalmology clinic demonstrated functional and anatomic improvement in both eyes. However, initial foveal involvement may leave photoreceptors defects that may limit the final visual outcome [23], this could explain the persistent rupture of Ellipsoid Zone in our patient after therapy. In the present case, cerebral MRI showed no enhancement of the optic nerve top or bottom, which also explains that radiological features are neither specific nor sensitive for ocular leukemia [24]. A recent review illustrated the poor sensitivity of CSF cytology [25]. Progress in the research and therapy of adult ALL is accelerating [26], and future studies should investigate better approaches for the early diagnosis of relapsed disease and more intensive strategies to improve patient survival.

In summary, understanding ocular involvement in leukemia is crucial since the eye is the only organ where leukemic infiltration to nerves and blood vessels can be observed directly. Recognizing fundus changes in leukemia allows earlier diagnosis and prompt treatment. Ophthalmologists should be aware of these possible manifestations and possible etiologies in patients with ALL, even if they have achieved disease remission. In this report, we present an unusual case of bilateral optic nerve infiltration and leukemic retinopathy as initial signs of Ph^+^-ALL relapse with CNS involvement in an elderly female and explore the underlying proposed pathologic basis of these ocular manifestations in leukemia. Hence, we highlight the priority and sensitivity of ophthalmic examinations, as they are noninvasive methods for detecting disease relapse.

## Data Availability

No datasets were generated or analysed during the current study.

## Abbreviations

Ph^+^-ALL: Philadelphia chromosome-positive acute lymphoblastic leukemia
CNS: Central nervous system
RE: Right eye
LE: Left eye
IOP: Intraocular pressure
OCT: Optical coherence tomography
MR: Magnetic resonance
CSF: Cerebrospinal fluid
BRB: Blood‒retinal barrier
